# *In vivo* Imaging of Glial Activation in Alzheimer's Disease

**DOI:** 10.3389/fneur.2018.00625

**Published:** 2018-08-07

**Authors:** Paul Edison, Cornelius K. Donat, Magdalena Sastre

**Affiliations:** Division of Brain Sciences, Department of Medicine, Imperial College London, London, United Kingdom

**Keywords:** inflammation, TSPO, Alzheimer's disease, positron emission tomography (PET), microglia, astrocyte, imaging

## Abstract

Alzheimer's disease (AD) is characterized by memory loss and decline of cognitive function, associated with progressive neurodegeneration. While neuropathological processes like amyloid plaques and tau neurofibrillary tangles have been linked to neuronal death in AD, the precise role of glial activation on disease progression is still debated. It was suggested that neuroinflammation could occur well ahead of amyloid deposition and may be responsible for clearing amyloid, having a neuroprotective effect; however, later in the disease, glial activation could become deleterious, contributing to neuronal toxicity. Recent genetic and preclinical studies suggest that the different activation states of microglia and astrocytes are complex, not as polarized as previously thought, and that the heterogeneity in their phenotype can switch during disease progression. In the last few years, novel imaging techniques e.g., new radiotracers for assessing glia activation using positron emission tomography and advanced magnetic resonance imaging technologies have emerged, allowing the correlation of neuro-inflammatory markers with cognitive decline, brain function and brain pathology *in vivo*. Here we review all new imaging technology in AD patients and animal models that has the potential to serve for early diagnosis of the disease, to monitor disease progression and to test the efficacy and the most effective time window for potential anti-inflammatory treatments.

## Introduction

Neuroinflammation is the term used to denote the response of the central nervous system to harmful stimuli such as protein aggregation, pathogens, and any other insult to the brain. While timely initiated and resolved, the inflammatory response is necessary reaction to noxious stimuli and hence protective, sustained and/or disproportionate (neuro)inflammation will likely contribute, exacerbate or induce tissue damage and thereby aggravate disease pathology (1). The nature of the inflammatory process is therefore complex and dynamic and changes along different stages of the disease, involving phenotypic alterations in all cells present within the CNS including neurons, microglia, astrocytes, and other inflammatory cells.

**Microglia**, act as part of the innate immune system, are constantly scanning and surveying the local microenvironment for signals of infection and injury [for a review see (2)]. Amyloid-β has been reported to “prime” or activate microglial cells (3). Different activation states were described in the past, so called “M1” or classically activated microglia or “M2” alternatively activated (2). The classically activated or pro-inflammatory phenotype has been associated with disease aggravation.

However, this classification has been recently challenged by single-cells transcriptomics, which suggests that the gene expression profile progressively switches with the disease and that this may even depend on how close they are to the amyloid plaques (4).

**Astrocytes**, are key mediators of many essential processes in the CNS. As for microglia, astrocytes have been classified into two distinct reactive states, A1 (inflammatory) and A2 (ischemic) states (5), although this classification seems to be over-simplistic. Accumulation of hypertrophic reactive astrocytes around senile plaques has been observed in post-mortem human tissue from AD patients [Reviewed by (6)] and in animal models of the disease (7). It is worth noting that astrocytes and microglia communicate with each other and this cross talk is important in promoting glial activation (8, 9).

In order to follow-up changes in microglial and astrocytic activation *in vivo*, radioactive tracers for positron emission and single-photon emission computed tomography (PET and SPECT) have been developed in the past decades. In this review, we will analyze different *in vivo* imaging techniques that allow the visualization of changes in neuroinflammation in animals and humans.

## *In vivo* imaging of microglia activation

### Pet imaging with TSPO ligands in AD patients

Following activation, microglia proliferate, and express a series of genes for pro-inflammatory cytokines and certain receptors on their surface, including the 18 kDa translocator protein (TSPO). TSPO, primarily but not exclusively expressed in the mitochondrial membrane of microglia, was previously identified as the peripheral-type benzodiazepine receptor (PBR) (10). Besides microglia, TSPO has been detected in other types of gliosis as well, such as in astrocytoma (11), and is generally expressed in highly proliferating cells. While the exact function of TSPO still remains to be elucidated, initially its role was associated with the transport of cholesterol, with the TSPO complex being a rate-limiting step in the synthesis of steroid hormones (12). Initial attempts to create TSPO knockout mice reported a non-viable phenotype [described in (13)]. However, the successful development of conditional TSPO knockout mice suggested that TSPO might not be a crucial part of steroid hormone synthesis, e.g., testosterone production (14). In addition, the mitochondrial expression-paradigm was challenged by reports of TSPO expression in other subcellular locations (15), e.g., nuclear/perinuclear-located TSPO, where is believed to play a part in cell proliferation (16). Moreover, plasma membrane bound TSPO has been observed as well, for instance in mature human red blood cells, lacking mitochondria (17, 18).

In the mammalian brain, the expression of TSPO turned out to be very low, compared to other tissues (19). Only the olfactory bulb and non-parenchymal regions, such as the ependymal and choroid plexus, showed higher TSPO densities in comparison with most gray and white matter structures (20, 21). However, under conditions of local inflammatory responses, e.g., caused by a multitude of brain injuries, neoplasms and infections, TSPO appears to be upregulated. This effect was quickly recognized and made TSPO a potentially ideal and sensitive biomarker of brain injury (10, 11, 22–25). Therefore, PET tracers for TSPO were developed in the past decade as markers for microglia activation and neuroinflammation in AD (26, 10) (see Table 1).

**Table 1:** *In vivo* imaging studies in patients with Alzheimer's disease or Mild Cognitive Impairment (MC) with the primary purpose of investigating neuroinflammatory changes through radiolabeled tracers.

**Radiotracer**	**Subjects used**	**Characteristics**	**References**
**18 kDa TRANSLOCATOR PROTEIN (TSPO)**			
**[**^**11**^**C]PK11195**	8 probable AD, “normal” elderly controls	No differences between controls and AD patients	(27)
	8 AD, 1 MCI, 15 HC	Increased tracer uptake in most brain regions	(28)
	13 AD, 14 HC	Increased tracer uptake in cortices, striatum and other regions, but not in hippocampus; positive correlation of tracer binding and cognitive scores, but not with [^**11**^C]PIB amyloid load	(29)
	10 AD, 10 HC	Higher average BP in AD subjects in neocortex and cerebellum; vascular binding different between AD and controls	(30)
	6 AD, 6 MCI, 6 HC	No differences between controls and MCI/AD patients	(31)
	22 AD, 14 MCI; 24 HC	Increased tracer binding in the frontal cortex of MCI subjects, coinciding with higher [^**11**^C]PIB amyloid load in the frontal cortex; no correlation between tracer binding and amyloid load or cognitive scores; tracer binding in AD patients not compared to MCI and controls	(32)
	11 AD, 4 HC	Increased tracer binding in cortices and cingulate but not in hippocampus; negative correlation of tracer BP and [^**11**^C]PIB amyloid load	(33)
	8 AD, 9 MCI, 17 HC	Comparison of different modeling approaches	(34)
	19 AD,10 MCI; 21 HC	No differences between groups in ROI analysis but small significantly increased clusters in voxel-wise comparison; no correlation of BP_ND_ and cognitive function	(35)
	8 AD, 15 HC	Increases in tracer binding in AD patients in several cortical regions, hippocampus and striatum with further mean increases at 16 months later; tracer binding coincided with increased [^**11**^C]PIB amyloid load and decreased [^**18**^F]FDG glucose metabolism	(36)
	9 PD dementia, 8 AD, 8 HC	Negative correlation between hippocampal volume and tracer binding	(37)
	8 MCI, 8 AD, 14 HC	MCI and AD patients showed an increased tracer binding as compared to control but reductions in tracer binding 14 months later	(38)
	42 MCI; 15 HC	85% of MCI patients showed increased tracer uptake in cortical regions; positive correlation of tracer uptake and [^**11**^C]PIB amyloid load in cortical regions; [^**11**^C]PIB positive MCI patients showed higher tracer binding; correlation of tracer binding with some cognitive scores	(39)
	16 PSP, 9 probable AD, 7 MCI; Part of the NIMROD study	Increased tracer uptake in most neocortical regions and putamen of AD patients; tracer binding correlates with cognitive scores	(40)
	
**[**^**123**^**I] iodo-PK11195 (SPECT)**	10 AD, 9 HC	Significant increase in tracer uptake in frontal and right mesotemporal cortices; significant negative correlation between cognitive scores and tracer uptake in several brain regions	(41)
**[**^**11**^**C] vinpocetine**	6 AD, 12 HC	No difference between AD and controls; Study limited by poor modeling and use of %SUV used as main outcome measure	(42)
**[**^**11**^**C] DPA-713**	7 AD, 12 healthy elderly and 12 healthy young subjects	Comparison with [^**11**^C]PK11195 binding; BP_ND_ levels significantly elevated in AD, but not for [^**11**^C](R)PK11195; Correlation of [^**11**^C]DPA-713 BP_ND_ levels and cognitive scores, again not observed for [^**11**^C]PK11195; no rs6971 genotyping	(43)
**[**^**18**^**F] DPA-714**	10 AD, 6 HC	No difference between AD and controls; no rs6971 genotyping	(44)
	9 probable-AD, 6 HC	significant differences in frontal and medial temporal lobes between subject groups in BP_ND_; no correlation between age, cognitive scores and disease duration; no rs6971 genotyping	(45)
	64 AD, 32 HC	rs6971 genotyping; higher tracer uptake in high and mixed affinity binders with AD compared to controls using volumes of interest and voxel-wise comparison, especially at the prodromal stage; tracer binding correlated with cognitive scores	(46)
**[**^**11**^**C] DAA1106**			
	10 AD and 10 HC	Mean BP increased in AD patients in all measured regions, significant in dorsal/medial prefrontal cortex, anterior cingulate cortex, striatum and cerebellum; no rs6971 genotyping	(47)
	10 MCI, 10 AD and 10 HC	Increased BP diffusely in MCI compared to control but no AD; No difference between AD and aMCI; no rs6971 genotyping	(48)
**[**^**18**^**F] FEDAA1106**	9 AD, 7 HC	No difference between AD and controls; no rs6971 genotyping;	(49)
**[**^**11**^**F] FEPPA**	21 probable AD, 21 HC	rs6971 genotyping; adjusted tracer binding significantly higher in AD in various gray and white matter areas; tracer binding was positively correlated with cognitive and sensory impairments	(50)
**[**^**11**^**F] FEMPA**	10 AD, 7 HC	rs6971 determined; increased V_T_ in the medial temporal cortex of high- and mixed affinity AD patients; High-affinity binding AD patients showed significantly higher tracer binding most investigated brain regions; correlation with cognitive scores	(51)
**[**^**11**^**C] PBR28**	10 MCI, 19 AD, 13 HC	rs6971 genotyping; No difference between MCI and healthy controls; AD patients showed widespread increases in cortical but not subcortical tracer binding as compared to MCI and controls, largely independent of genotype; tracer binding was significantly negatively correlated with several cognitive scores, gray matter volume and [^**11**^C]PIB amyloid burden	(52)
	9 MCI, 6 AD and 7 HC	Association of higher [^**11**^C]PBR28 SUV with poly (ADPribose) polymerase 1 polymorphisms in subjects at risk for AD	(53)
	11 MCI, 25 AD and 21 HC	All patients [^**11**^C]PIB positive and rs6971 genotyped; tracer V_T_ higher temporal and parietal cortex in MCI/AD; cerebellum a suitable pseudo-reference region not significantly different among the three groups in cerebellum	(54)
	14 probable MCI/AD, 8 HC	[^**11**^C]PIB positive patients (rs6971 genotyped) had higher tracer binding in cortical regions and hippocampus and showed a 2.5–7.7% annual increase in follow-up scans; tracer binding was correlated with disease progression, measured as worsening of cognitive scores	(55)
	11 PCA, 11 AD, 15 HC	Higher tracer retention of PCA patients in occipital, posterior parietal, and temporal regions, AD patients with increased tracer binding in inferior and medial temporal cortex; tracer binding correlated with cortical volume loss and reduced glucose metabolism	(56)
	21 AD and 15 HC	BP_ND_ can be estimated without arterial input function and shows similar effect sizes in AD patients compared to arterial input function derived data in AD patients	(57)
**MONOAMINE OXIDASE B - [**^**11**^**C]DEUTERIUM-L-DEPRENYL**			
**[**^**11**^**C]deuterium-L-deprenyl**	9 AD, 11 HC	Significantly increased tracer retention after blood-flow corrections in frontal, parietal and temporal cortex of AD patients, No difference in sensorimotor, occipital cortex and subcortical regions; significant correlation of tracer binding and [^**11**^C]PIB amyloid load	(58)
	8 MCI, 7 AD, 14 HC	Significant increase in tracer binding in bilateral frontal and parietal cortices in MCI and AD patients; no change in subcortical regions; no correlation of the tracer binding with [^**11**^C]PIB amyloid load or [^**18**^F]FDG glucose metabolism	(59)
	17 MCI, 8 AD	Significant correlation between tracer binding and [^**11**^C]PIB amyloid burden in AD; MCI [^**11**^C]PIB positive patients show higher tracer binding in parahippocampus	(60)
	17 MCI, 8 AD, 11 HC	Tracer binding found to be independent of brain perfusion and able to discriminate between groups	(61)
**CALCIUM INFLUX USING** ^**57**^**CO SPECT**			
^**57**^**Co (SPECT)**	6 probable AD, 5 vascular/fronto-temporal dementia	No specific uptake patterns in probable AD patients as compared to other dementia patients	(62)

*AD, Alzheimer's disease; HC, Healthy controls; MCI, Mild Cognitive Impairment; PCA, posterior cortical trophy; PD, Parkinson's disease; PSP, progressive supranuclear palsy*.

The first and probably most widely used TSPO radiotracers, with over thousand publications, are the antagonist PK11195 [1-(2-chlorophenyl)-N-methyl-N-(1-methylpropyl)-3-isoquinoline carboxamide] and the agonist Ro5-4864 (63). Initial studies with PK11195 were conducted more than two decades ago, with numerous subsequent papers demonstrating upregulation in different neurodegenerative diseases and in neuroinflammatory conditions (64–67). Unsurprisingly, increased TSPO expression was reported by autoradiography in a wide range of brain regions of post-mortem samples from AD patients, including hippocampus, frontal, temporal, and parietal cortices (22, 68, 69).

Cagnin et al. published the first PET study using [^11^C]PK11195 in 2001, demonstrating an increase of tracer uptake in AD cases (28). While subsequent reports generally have shown increased tracer binding in AD brains, some publications found no differences between Alzheimer's subjects and healthy individuals (29, 33, 35). In spite of these conflicting results, it is generally accepted that there is increased microglial activation in AD. Recent studies have extended this to patients with Mild cognitive impairment (MCI), showing that glia activation can precede clinical AD (70).

Recent reports have evaluated the relationship between amyloid load and neuroinflammation, suggesting that microglial activation is associated with amyloid load. Interestingly, using [^11^C]PK11195 and the amyloid tracer [^11^C]PIB, one study did not show any correlation between the binding of these tracers, while another one suggested a negative correlation between amyloid-β and TSPO density (31, 33). The reason behind these different outcomes could be related to the limitations of the current amyloid ligands; while we are able to image amyloid plaque deposition using amyloid imaging agents, other forms of amyloid, such as β-amyloid oligomers, which may be contributing to the microglial activation, are not currently detectable by PET (36, 71).

**Figure 1:**
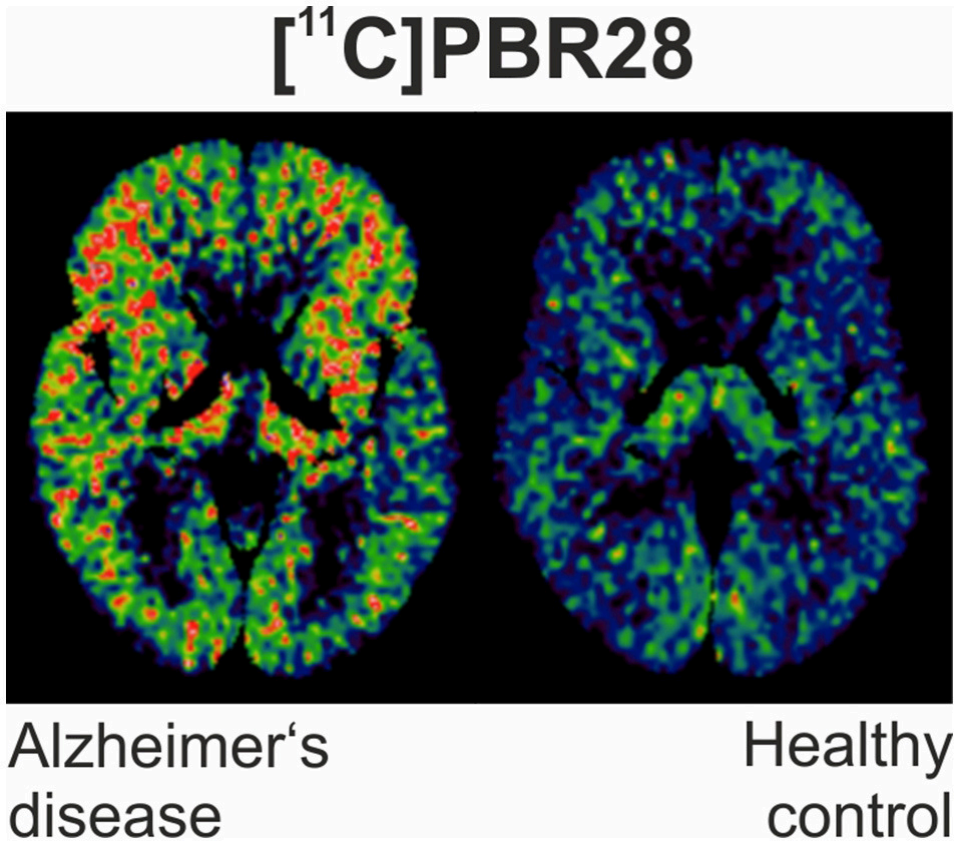
[^11^C]PBR28 binding is significantly increased in different cortical regions in an Alzheimer's disease subject (MMSE of 22/30) compared to healthy control (MMSE 30/30).

However, rigorous quantification of TSPO density using [^11^C]PK11195 has been confronted by limitations of the ligand, including its modest binding affinity, high non-specific binding and elevated lipophilicity, generating a low signal-to-noise ratio (72). This has led to the development of numerous “second-generation“ TSPO ligands [^11^C]AC-5216, [^18^F]PBR111, both ^11^C and ^18^F radiolabeled derivatives of PBR06 and PBR28, [^18^F]FEPPA, [^18^F]DPA-714 and the SPECT tracer [^123^I]CLINDE (46, 48, 64, 73–79). Figure 1 illustrates an example of PET imaging with [^11^C]-PBR28 showing increased binding in an AD patient.

However, while affinity and nonspecific binding properties were usually found to be improved as compared to PK11195, it quickly became apparent that these new TSPO tracers are affected by genetic variability of TSPO binding site induced by the rs6971 single-nucleotide polymorphism (80), resulting in high-, mixed and low-affinity binders. This polymorphism restricts studies with these tracers to high- and mixed-affinity binders. Recently, “third-generation” TSPO tracers, such as GE-180 and ER176 (81, 82) were developed and aimed at allowing TSPO quantification regardless of rs6971 genotype, however with mixed success and no published data in AD patients. Additional considerations to take into account when using these ligands *in vivo* are the different modeling approaches and reference regions (83), along with other methodological issues reviewed by Donat et al. (84).

In order to follow up changes of TSPO over time, longitudinal studies in AD have recently been published. Two studies (36, 38) revealed reductions in [^11^C]PK11195 binding in MCI patients with different amyloidosis status, whereas increased binding was found in diagnosed AD patients. A moderate increase of [^11^C]PBR28 uptake in 14 patients with AD was associated with worsening clinical symptoms (55). The most recent longitudinal study with [^18^F]DPA-714 demonstrated that prodromal and demented AD patients display an initially higher TSPO density as compared to controls. However, when classifying patients into slow and fast decliners according to functional (Clinical Dementia Rating change) or cognitive (Mini-Mental State Examination score decline) outcomes, it was shown that slow decliners show a higher initial [^18^F]DPA-714 binding than fast decliners, suggesting that higher initial [^18^F]DPA-714 binding is associated with better clinical prognosis (85).

### Pet imaging in animal models of AD

The first studies carried out in animal models of amyloidosis demonstrated a significant age-dependent increase in the specific binding of [^3^H]PK11195 in the TASTPM model (APPswxPS1M146V) by autoradiography (8), in agreement with age-dependent increases in CD68 immunoreactivity co-localized with Aβ deposits. However, reports on [^11^C]PK11195 PET imaging in mouse models of amyloidosis have exposed conflicting results, depending on the model used and age of the animals. A higher [^11^C]PK11195 uptake was shown in the brains of older APP/PS1 mice when compared with age-matched controls (68, 86). Surprisingly, [^11^C]PK11195 binding in younger transgenic APP/PS1 mice was not different from their controls, even though immunostaining revealed activated microglia in close proximity of amyloid deposits. Similar to human data, it is likely that different modeling approaches and reference regions may contribute to the seemingly conflicting *in vivo* findings.

Our own recent autoradiographic and PET data provided evidence of an increased binding of [^3^H]PBR28 in the brain of the aggressive 5XFAD mouse model, compared with wild-type controls, which coincided with the strongly increased immunoreactivity of the microglial marker Iba1 in the same brain areas (87). These results provided support for the suitability of PBR28 as a tool for monitoring of (micro)-glial activation. [^3^H]PBR28 binding was significantly higher in female animals and positively correlated between Aβ plaque load and tracer binding. In addition, using [^11^C]PBR28 in healthy rats, *in vitro* brain autoradiography showed a 19% increase of binding in aged (19.6 months) as compared in young rats (4 months) (88).

Besides PBR28, other new generation tracers have exhibited similar patterns in animal models of AD. Increases in TSPO density were reported from 10 months old Thy1-hAPPLond/Swe (APPL/S) mice compared with wild-type controls, using *ex vivo* autoradiography with [^18^F]PBR06, but this increase was only observed in older mice, at 16 months of age by PET (89). Similar findings were published by Liu et al. (90), who performed [^18^F]GE180 PET in young and old wild-types (WTs) and APP/PS1dE9 transgenic mice, showing higher uptake in transgenic and WT mice at 24 months of age but not in young 4 months old transgenics (90). In a different study, [^18^F]GE180 uptake was slightly increased in PS2APP mice at 5 mo and markedly elevated at 16 mo. Over this age range, there was a highly positive correlation between TSPO PET uptake, amyloid load and likewise with tracers for brain metabolism (91). However, a recent study in APP23 mice showed that the increased rate of (micro)glia activation detected with [^18^F]GE-180 appears to be of less magnitude than the elevation in amyloidosis detected with [^11^C]PIB over time. In fact, [^18^F]GE-180 binding seems to plateau at an earlier stage of pathogenesis, whereas amyloidosis continues to increase. These results suggest that TSPO might be a good marker for early pathogenesis detection, but not for tracking long-term disease progression (92).

These tracers have also served to assess and monitor the efficacy of anti-inflammatory treatments. LM11A-31 is a p75 neurotrophin receptor ligand that was shown to reduce the hyperphosphorylation and misfolding of tau, decrease neurite degeneration, and attenuate microglial activation. LM11A-31-treated APPL/S mice displayed significantly lower [^18^F]GE-180 binding in cortex and hippocampus of as compared to vehicle-treated animals, corresponding to decreased TSPO and Iba1 staining (93).

As AD is characterized by substantial aggregation of hyperphosphorylated tau, second-generation TSPO ligands have also been employed in transgenic models of tau pathology, such as the PS19 mice. Here, uptake of [^11^C]AC-5216 was found linearly proportional to the phospho-tau immunolabelling (94).

While TSPO is the most widely recognized biomarker of neuroinflammations, other targets have been explored in recent years. Radiolabeled ketoprofen methyl ester, [^11^C]-KTP-Me is a highly selective tracer for the cyclooxygenase-1 (COX-1). In APP transgenic mice, [^11^C]-KTP-Me uptake was significantly increased in the brain of 16 to 24 mo old mice in comparison to their age-matched controls, coinciding with the histopathologic appearance of abundant Aβ plaques and activated microglia. Furthermore, [^11^C]-KTP-Me accumulation was observed in the frontal cortex and hippocampus, whereby COX-1-expressing activated microglia appeared surrounding Aβ plaques, indicating neuroinflammation that originated with Aβ deposition (95). Another currently investigated alternative to TSPO ligands are tracers for the cannabinoid 2 receptor, such as [^11^C]Sch225336 and [^11^C]A-836339 (96–98) and tracers for the purinergic receptors P2Y12 and P2X7 (99).

### Magnetic resonance spectroscopy (MRS)

Magnetic resonance spectroscopy (MRS) is a new technique that can provide information about several relevant metabolites for neuroinflammation and neurodegeneration. Recently, chemical exchange saturation transfer (CEST), as a novel molecular MR imaging approach, has been developed, which uses proton exchange as a means of enhancing the contrast of specific molecules in the body (100). Endogenous CEST compounds include hydroxyl (OH), amine (NH2), and amide groups (NH). In the last few years, several studies have explored the possibility of imaging neuroinflammatory and neurodegeneration biomarkers *in vivo* with CEST, such as CEST imaging of myo-inositol (101), glutamate (102) and glucose (103) in AD mouse models.

## *in vivo* imaging of astrocytes

### Pet imaging of astrocytes in humans

The best-known tracer for astrocytes so far is [^11^C]deuterium-L-deprenyl [[^11^C]DED], which is an irreversible monoamine oxidase B (MAO-B) inhibitor. This is based on previous findings showing that astrocytes express elevated levels of MAO-B during their activation. The ligand has been therefore employed as biomarker of astrocytosis in pathologies such as AD (59) and amyotrophic lateral sclerosis (ALS) (104). Increased [^11^C]DED binding throughout the brain was detected in MCI [^11^C]PIB-positive patients compared with controls and MCI [^11^C]PIB-negative and AD patients (59). In autosomal dominant AD carriers, astrocytosis measured by [^11^C]DED was found initially high and then declining, contrasting with the increasing amyloid-β plaque load during disease progression, suggesting astrocyte activation is implicated in the early stages of AD pathology (61).

In the last years, new ligands for the potential imaging of astrocytes have been developed, including those for type-2 imidazoline receptors (I2Rs), which were found to be expressed primarily in astrocytes. These receptors were described for the first time in the 90's and the first studies performing *in vitro* binding with [^3^H]idazoxan in postmortem cortical membranes showed increased density in AD patients (105). Later on, work carried out with the I2R PET tracer [^11^C]FTIMD reported specific binding to these receptors in rat and monkey brains, but exhibiting a relative low binding specificity (106, 107). More recently, [^11^C]BU99008 (2-(4,5-Dihydro-1H-imidazol-2-yl)-1- methyl-1H-indole) was developed as a more potent PET ligand for I2Rs imaging (108, 109), displaying relatively high binding specificity and brain penetration in the porcine and rhesus monkey brain (108, 110). There are ongoing human PET imaging trials in Alzheimer's and healthy control patients at the moment using [^11^C]BU99008 and the preliminary results have shown good brain delivery, reversible kinetics, heterogeneous distribution specific binding signal consistent with I2BS distribution and good test-retest reliability (111).

### Imaging of astrocytes in models of AD

#### Pet imaging for astrocytes

Imaging studies with [^11^C]DED carried out in transgenic APP Swedish (APPswe) mice and wild–type animals at different ages, have demonstrated that tracer uptake was significantly higher at 6 months than at 18–24 months in APPswe mice, preceding Aβ deposition (112). However, no differences in [^3^H]-L-deprenyl obtained by autoradiography were observed between WT and APPswe mice across different ages. Furthermore, staining of the astrocyte marker GFAP was increased in older transgenic APPSwe mice as compared to younger mice (112), raising questions about the specificity of this ligand as marker for astrocytes.

#### *in vivo* bioluminescence imaging (BPI)

Bioluminescence describes the light produced by the enzymatic reaction of a luciferase with its substrate (luciferin) and the emitted light is detected with a camera. The technique allows for fast acquisition times so that subjects can be imaged quickly, serially over time and with minimal distress. The Prusiner's lab developed *in vivo* bioluminescence imaging and quantitative determination of inflammation in a model of prion related neurodegenerative disease (113). Additionally, bigenic mice overexpressing APP and GFAP-Luc were reported to show an age-dependent increase in signal that was corresponded to major areas of Aβ deposition. Bioluminescence signals began to increase in 7-mo-old Tg(CRND8:Gfap-luc) mice and at 14-mo-old in Tg(APP23:Gfap-luc) mice (114).

Gfap-luciferase reporter mice have also been crossed-bred with hTau40AT/C57BL/6N mice. *In vivo* bioluminescence imaging (BLI) showed activation of astrocytes in response to aggregation of Tau, from 5 months of age compared with wild-type animals (115).

## Conclusions and future perspectives

The development of new neuroinflammation tracers in the last decade has allowed characterizing the pattern of glia activation in AD patients, showing that it occurs ahead of amyloid deposition, correlates in many cases with amyloid plaque density and allows limited predictions of disease progression. The longitudinal studies have shown that this glial activation, as detected by PET, fluctuates during disease progression. Although reports in animal models of AD have helped confirming the specificity of TSPO tracers for microglia, the situation is not the same for tracers for astrocytes and more research needs to be done regarding this aspect. In addition, new tracers able to differentiate between the potential M1 and M2 microglial phenotypes would be advantageous in identifying their function *in vivo* (116).

Future studies should include imaging in patients after intervention with anti-inflammatory drugs; however so far, there are no reports in that aspect in AD cases. Therefore, imaging studies are key to test the efficacy and the most effective time window for potential anti-inflammatory treatments.

## Author contributions

PE and MS designed this review outline, PE performed most of the literature review on TSPO imaging in humans, CD edited and the manuscript, performed the literature review for the table and MS wrote the rest of the manuscript.

### Conflict of interest statement

The authors declare that the research was conducted in the absence of any commercial or financial relationships that could be construed as a potential conflict of interest.
